# Identification of Candidate Polymorphisms on Stress Oxidative and DNA Damage Repair Genes Related with Clinical Outcome in Breast Cancer Patients

**DOI:** 10.3390/ijms131216500

**Published:** 2012-12-05

**Authors:** Patricia Rodrigues, Jessica Furriol, Begoña Bermejo, Felipe Javier Chaves, Ana Lluch, Pilar Eroles

**Affiliations:** 1 Institute of Health Research INCLIVA, Av. Blasco Ibañez, 17, Valencia 46010, Spain; E-Mails: patriciaels@gmail.com (P.R.); Jessica.Furriol@uv.es (J.F.); lluch_ana@gva.es (A.L.); 2 Department of Haematology and Medical Oncology. Valencia University Clinical Hospital, Valencia 46010, Spain; E-Mail: begobermejo@gmail.com; 3 Genotyping and Genetic Diagnosis Unit, Valencia University Clinical Hospital, Institute of Health Research INCLIVA, Valencia 46010, Spain; E-Mail: felipe.chaves@uv.es

**Keywords:** genetic variants, GCLC, XDH, OGG1, breast cancer, survival

## Abstract

Diverse polymorphisms have been associated with the predisposition to develop cancer. On fewer occasions, they have been related to the evolution of the disease and to different responses to treatment. Previous studies of our group have associated polymorphisms on genes related to oxidative stress (rs3736729 on *GCLC* and rs207454 on *XDH*) and DNA damage repair (rs1052133 on *OGG1*) with a predisposition to develop breast cancer. In the present work, we have evaluated the hypothesis that these polymorphisms also play a role in a patient’s survival. A population-based cohort study of 470 women diagnosed with primary breast cancer and a median follow up of 52.44 months was conducted to examine the disease-free and overall survival in rs3736729, rs207454 and rs1052133 genetic variants. Adjusted Cox regression analysis was used to that end. The Kaplan-Meier analysis shows that rs3736729 on *GCLC* presents a significant association with disease-free survival and overall survival. The polymorphisms rs1052133 on *OGG1* and rs207454 on *XDH* show a trend of association with overall survival. The analysis based on hormonal receptor status revealed a stronger association. The CC genotype on rs207454 (*XDH*) was significantly associated with lower time of disease free survival (*p* = 0.024) in progesterone receptor negative (PGR−) patients and rs3736729 (*GCLC*) was significantly associated with disease free survival (*p* = 0.001) and overall survival (*p* = 0.012) in the subgroup of estrogen receptor negative (ER−) patients. This work suggests that unfavorable genetic variants in the rs207454 (*XDH*) and rs3736729 (*GCLC*) polymorphisms may act as predictors of the outcome in negative progesterone receptor and negative estrogen receptor breast cancer patients, respectively.

## 1. Introduction

Breast cancer is one of the most common cancers among women worldwide. New treatments and early diagnoses have contributed substantially to improve the survival rate of patients [1,2]. Regardless, novel biomarkers are needed to identify subgroups according to their estimated prognosis. It has been established that polymorphism variants on low penetrance genes can contribute to the risk of breast cancer [3–11]. Equally so, in certain cases, an association between these genetic variants and the progression of the disease has been found [12–20]. Still, the degree of oxidative stress has been linked to the development of breast cancer [21]. Reactive oxygen species (ROS) are the natural product of respiration and other normal cellular processes. These species have been shown to induce cell death by causing different types of cellular damage associated with lipid peroxidation and alterations of nucleic acids and proteins [22], triggering apoptosis through the mitochondria [23]. Excess oxidative stress as a consequence of the alteration of the balance between reactive oxygen species (ROS) and antioxidant enzymes may lead to cellular apoptosis [24], proliferation and/or tumor promotion [25]. Consistently, common variants in genes related to the stress pathway and DNA damage repair genes have been good candidates for cancer susceptibility and prognosis.

Antioxidant enzymes, such as catalase, superoxide dismutases, glutathione peroxidases, glutathione reductase and thioredoxins, have been shown to protect from oxidative damage. Hydroxyl radical interaction with DNA creates different types of oxidized nucleoside. 8-OHdG is one of the most commonly occurring of these DNA modifications. 8-Oxodeoxyguanosine lesions can be excised by 8-oxoguanine DNA glycolase, the enzyme encoded by *OGG1*[26–28]. We, among others authors, have found that the homozygote genotype for the infrequent allele (G) in the polymorphism rs1052133 on this gene confers an increment of risk to developing breast cancer (OR = 1.82 (95% CI, 1.31–2.52) and *p*-value = 0.0004) [29].

Furthermore, genetic variants of genes that generate cellular ROS can influence the final oxidative stress balance. These enzymes include, among others, xanthine dehydrogenase (XDH), NO synthases, NADPH oxidases (NOXs) and mitochondrial oxidases. XDH belongs to the group of molybdenum-containing hydroxylases involved in the oxidative metabolism of purines [30]. The rs207454 polymorphisms on this gene present an association with breast cancer in our previous studies (OR = 2.12 (95% CI, 1.11–4.04) and *p*-value = 0.024). It predisposes to the illness, in a recessive model.

The enzymes of the glutathione system are important for a variety of biological functions, including the protection of cells from oxidative damage by free radicals, detoxification of xenobiotics and membrane transport. GLCL is one of the most important intracellular antioxidants participating in the detoxification reactions of several cytotoxic drugs [31]. The glutamate-cysteine ligase is the first rate-limiting enzyme of glutathione synthesis. The enzyme consists of two subunits, a heavy catalytic subunit (GCLC) and a light regulatory subunit (GCLM). We have found an association of the polymorphism rs3736729 on the *GCLC* gene with breast cancer (OR = 0.85 (95% CI, 0.73–1.00) and *p*-value = 0.054) in a recessive model.

As shown above, previous data from our group relate the polymorphisms rs1052133 on the *OGG1* gene, rs207454 on the *XDH* gene and rs3736729 on the *GCLC* gene to susceptibility to breast cancer. The aim of the present study was to investigate the possible association of these polymorphisms with overall and relapse-free survival of breast cancer patients. To that end, we performed a survival analysis based on genetic variants in a group of patients with primary breast cancer diagnoses and with available follow-up information.

## 2. Results and Discussion

### 2.1. Survival Analysis

We analyzed the survival curves for the three polymorphisms in a group of 470 breast cancer patients with follow up. The characteristics of these patients are detailed in Table 1. Of the 470 patients included in the study, 159 patients progressed and 88 died. The Kaplan-Meier analysis shows a trend to association with overall survival (OS) for the polymorphisms rs1052133 on the *OGG1* gene and rs207454 on the *XDH* gene.

**Figure 1:**
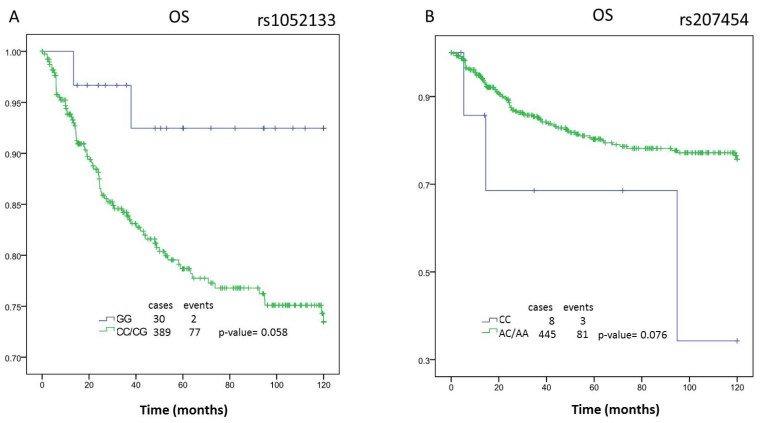
Kaplan-Meier analysis of rs1052133 and rs207454 polymorphisms. **A** and **B**: overall survival (OS) of total patients in function of the polymorphisms rs1052133 and rs207454, respectively.

The survival analysis for rs1052133 shows a trend (*p*-value = 0.058, borderline significance) with an OS increase of 15.02 months in the patients with genotype CC + CG *versus* the patients with the GG genotype (CC + CG: 113.00 months [95% CI 103.64 to 122.35 months] *vs.* GG: 97.98 months [95% CI 93.52 to 102.45 months]) (Figure 1A). Similarly, the polymorphisms rs207454 were associated with the risk of breast cancer in a recessive model. The genotype CC predisposes to the illness, and it also shows a lower overall survival in the Kaplan-Meier analysis (AA + AC: 99.67 months [95% CI, 95.62 to 103.72 months] *vs.* CC: 76.90 months [95% CI 39.60 to 114.20 months]) with a difference between medians of 22.77 months (*p* = 0.076) (Figure 1B).

The polymorphism rs3736729 on the *GCLC* gene shows a significant association with the DFS and OS (Table 2). If we compare the three possible genotypes (CC, AA and AC) with the time to first recurrence, the association is statistically significant (*p*-value = 0.015). The AC genotype shows a lower DFS median time compared to the other genotypes (AC: 78.62 months [95% CI, 71.07 to 86.17 months] *vs.* AA: 90.74 months [95% CI, 82.74 to 98.74 months] and CC: 93.24 months [95% CI, 83.98 to 102.49 months]). The association is even more powerful (*p* = 0.004) when we compare the heterozygote *vs.* homozygote genotypes (AC: 78.62 months [95% CI, 71. 07 to 86.17 months] *vs.* AA/CC: 91.77 months [95% CI, 85.71 to 97.82 months]). The analysis of OS shows a similar tendency with a borderline significance (*p* = 0.05). We did not find any relationship between the drug or administration treatment followed by the patients and the outcome. The analysis of association between polymorphisms and the characteristic of the patients shows significance for the tumor size and the hormonal receptor status (data not shown). Consequently, we performed the analysis of survival depending on these variables.

**Figure 2:**
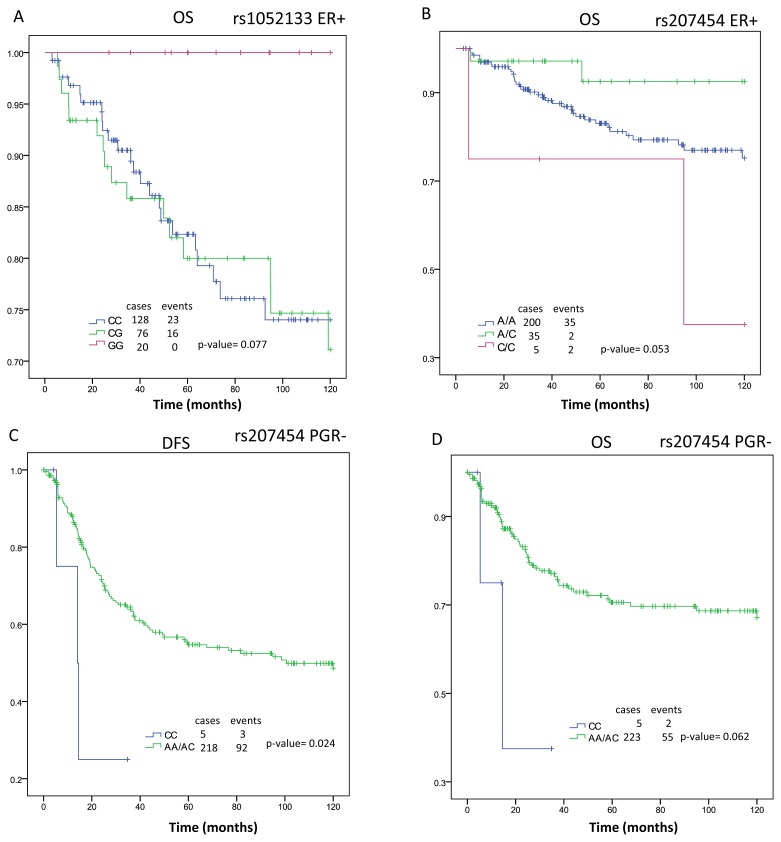
Kaplan-Meier analysis of rs1052133 and rs207454 polymorphisms. **A** and **B**: overall survival in the subgroup of estrogen receptor positive (ER+) patients in function of the polymorphisms rs1052133 and rs207454, respectively; **C**: Diseases-free survival (DFS) and **D**: overall survival (OS) in the subgroup of progesterone receptor negative (PGR−) patients in function of the polymorphism rs207454.

The polymorphisms rs1052133 and rs207454 show a trend toward significance in the OS analysis when the patients were ER+ (*p* = 0.077 and *p* = 0.053, respectively) (Figure 2A,B). The analysis, as a result of the progesterone receptor (PGR) status, shows an association only in the case of the rs207454, with a significant value in the time of DFS (*p*-value = 0.024) and a trend in the OS (*p* = 0.062). Both cases present the lowest survival time in patients with the CC genotype and PGR− (Figure 2C,D).

**Figure 3:**
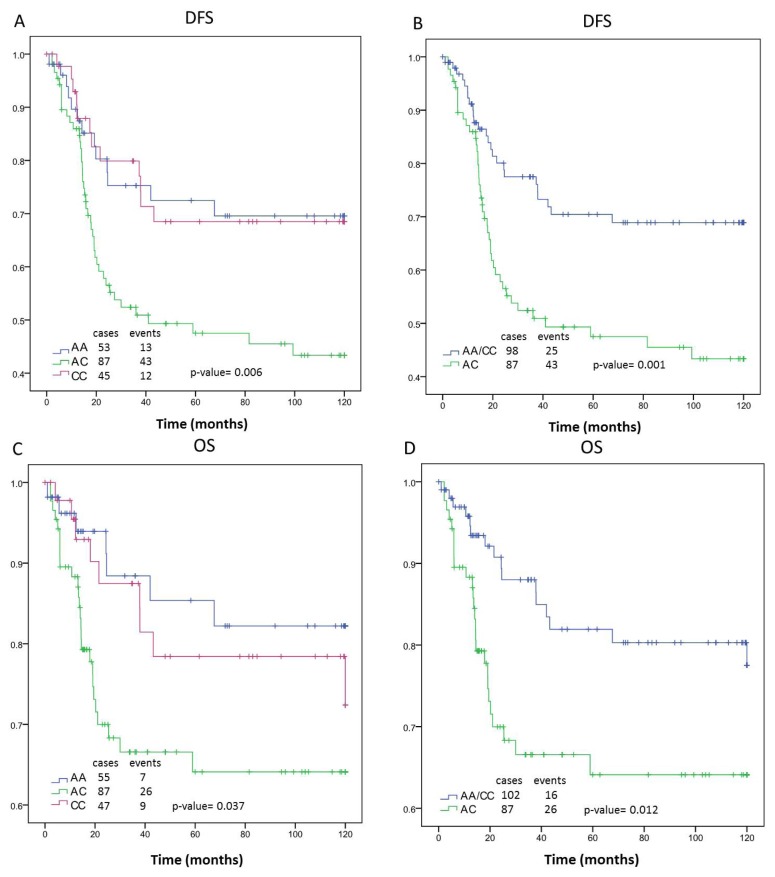
Kaplan-Meier curves of rs3736729 showing disease-free survival (DFS) overall survival (OS) of estrogen receptor negative (ER−) breast cancer patients. **A** and **C**: rs3736729 genotypes A/A, A/C and C/C; **B** and **D**: rs3736729 genotypes A/A and C/C *vs.* A/C; **A** and **B**: DFS; **C** and **D**: OS.

The genotype of the polymorphism rs3736729 presents the highest association with ER− patients in comparison to the total breast cancer cases analyzed. The data for DFS showed that the patients with the AC genotype, and in the absence of ER expression, present the worse median survival (65.01 months [95% CI, 53.59 to 76.43 months], *p*-value = 0.001) compared to patients with the other genotypes. The difference in survival time between these groups is 24.62 months. Consequently, the OS was reduced in the ER− *vs.* overall group for the AC genotype (82.99 months [95% CI, 71.32 to 94.66 months] *vs.* 93.02 months [95% CI, 85.77 to 100.26 months]). The decreased survival is 18.64 months. Figure 3 shows the survival curves according to the *GLCL* genotype of ER− patients. There were no significant associations between genotype and either DFS or OS among patients with ER+. The ten year DFS and OS for each rs3736729 genotype by tumor ER and PGR status and overall group are shown in Table 2.

The analysis of association between PGR and rs3736729 shows a relationship exclusively with the PGR− subgroup of patients, where the AC genotype again corresponds to the worst outcome (64.82 months [95% CI, 53.97 to 75.67 months]. However, the significance is not as good as that for the ER− subgroup (*p* = 0.001 *vs. p* = 0.032, respectively). The combination of PGR− and ER− shows a DFS median survival with lower significance than for ER− alone (*p* = 0.005 *vs. p* = 0.001, respectively). These data suggest a possible contribution of the AC genotype to DFS in patients according to their ER status. A similar conclusion may be reached in the OS study for ER− and PGR− *vs.* ER−(*p* = 0.017 *vs. p* = 0.012, respectively). The analysis of the polymorphism rs1052133, rs207454 and rs3736729 with the tumor size does not show a significant relationship.

### 2.2. Discussion

The influence of many polymorphisms in the predisposition to breast cancer has been described in the literature. However, the relationship between these genetic variants and outcomes in breast cancer patients is more limited. To best understand the role of the three significant SNPs in breast cancer, in this work, we decided to analyze the correlation of breast cancer patient survival with the different genotypes.

Our results suggest an association between the rs207454 polymorphism on *XDH* (encoding an enzyme involved in the oxidative metabolism of purines) [32] and survival of breast cancer patients. The OS in the total population shows a trend toward significance. However, the analysis in the subgroup of PGR patients presents a significant association between the genotype and the time to relapse of the illness when using a recessive model (AA + AC *vs.* CC). These data suggest that the rs207454 genotype could be a marker of DFS in the subgroup of patients in which PGR is absent.

Our data suggest an association between the polymorphisms rs3736729 on the *GCLC* gene and the progression of breast cancer patients compatible with an over-dominant model, where the genotype AC gives the worst prognosis in patients with breast cancer. These results indicate that individuals heterozygous for this polymorphism have a remarkably poor prognosis *vis-à-vis* the evolution of their breast cancer. The most common models adopted in statistics are the dominant and the recessive ones. Nevertheless, over-dominance is observed in many human diseases. Molecular heterosis is common in humans and may occur in up to 50% of gene associations. Heterosis in the *SLC6A4* serotonin transporter gene, *HTR2A*, *ESR1*, *TIGR* and *DRD2* genes has been described. A possible mechanism by which heterosis could exert a biological effect is at the level of protein subunit interaction. The heterosis could produce allosteric changes that affect protein function and result in a more (or less) efficient protein than the wild-type [33]. Our hypothesis is that the heterozygote genotype of rs3736729 could decrease the efficacy of the GLCL enzyme and, as a result, diminish the antioxidant capacity of each person.

The sharp association between the rs3736729 genotype on *GCLC* and the absence of ER suggest the existence of a possible mechanism of interrelation between the nuclear receptor and genes of the stress oxidative pathway. Indeed, 17-β-estradiol induces production of antioxidative enzymes, including gamma-glutamylcysteine synthetase (g-GCS/GCLC). The effects of 17-β-estradiol are mediated mostly through ER, which belong to the nuclear receptor superfamily and functions as a ligand-induced transcription factor. Therefore, one of the important ways to increase the expression of GCLC is through the activation of the ER receptor. Furthermore, the enzyme GCLC, which plays a critical function in the protection against reactive oxygen species, is also essential in the process of detoxification of xenobiotics. The exposure of cells to these agents results in a significant increase of the enzyme levels by the transcriptional up-regulation of the genes (*GCLC* and *GLCM*) encoding the two subunits [34,35]. This bibliographic evidence suggests that ER− patients would have less anti-oxidative potential as a consequence of the ER expression reduction. Consequently, the expression levels of GCLC induced by ER would decrease. The combination of this fact with a possible unfavorable rs3736729 genotype (AC) could potentiate the early recidive of this group of patients. This hypothesis seems reasonable, since several studies suggest that oxidative stress contributes significantly to cancer progression by the alteration of the redox control of the cell cycle [36,37]. We postulate that the genotype of rs3736729 could determine different outcomes in ER− breast cancer patients, just as the genotype of rs207454 could influence the time of disease free survival in PGR− patients. This is critical to the identification of subpopulations for which the current therapy is insufficient, and new clinical approximations are required. Several therapeutic agents commonly used in a clinical setting, such as radiation therapy and chemotherapeutic agents, exert antitumor effects through the increased formation of reactive oxygen species (ROS). Nowadays there are in development pharmacological agents (considered dirty drugs) that modulate cellular redox through pleiotropic interactions. Ongoing studies are testing the activity of these agents as combinatorial drugs consistent with the induction of deviation from the redox homeostasis that sensitizes cancer cells to the cytotoxic effects of chemotherapeutic agents. The benefit provided by redox chemotherapeutics depends on the genotypic and phenotypic profiling of the patients, as the case may be for the different rs3736729 or rs207454 genotypes.

Our study has several potential limitations to consider, such as the possible interaction with other polymorphisms located in these genes, but not included in this study. Also, some SNPs are located in non-coding regions. However, variations in intronic structure have been proposed as an influence in cancer via the regulation of gene expression, gene splicing or mRNA stability. It is also possible that these polymorphisms are in linkage disequilibrium with other functional polymorphisms that may affect breast cancer outcome. Despite these considerations, our work, as far as we know, is the first study that shows a relationship between polymorphisms on the *GCLC* and *XDH* genes and the survival outcome in ER− and PGR− breast cancer patients, respectively. The results obtained in this work seem to sustain the hypothesis of the participation of oxidant and antioxidant genes in breast cancer. However, we consider further studies necessary to confirm this result in independent population groups and to test for possible therapeutic alternatives.

## 3. Experimental Section

### 3.1. Study Population

The present study included a population-based cohort of 470 women diagnosed with primary breast cancer at the Clinic Hospital of Valencia (Spain). The recruitment was done between 1983 and 1998, and the subjects were adult, Caucasian females (mean age at diagnosis of 54.1 years, range 20.5–86.5) and residents in the area of influence of the Hospital. Recurrences were determined by oncologists, and patient death was always confirmed by death certificates. All the participants in the study were informed and gave their written informed consent to participate in the study. The characteristics and follow-up of patients was routinely updated in the data bases by trained Hospital personnel. The mean follow-up was 52.44 months.

### 3.2. DNA Extraction and Genotyping

The blood samples of the 470 patients were collected over a period of six months prior to the start of this study. Genomic DNA was extracted from blood samples using the DNeasy tissue kit from Qiagen (Izasa, Madrid, Spain). A final elution volume of 100 μL was established. DNA quantity was measured by absorbance at 260nm using a NanoDrop spectrophotometer, and DNA purity was evaluated by measurement of the 260/280 absorbance ratios. Each DNA sample was stored at −20 °C until analysis. Genotyping analysis was performed by SNPlex (genotyping system based on ligation assay/polymerase chain reaction technology; Applied Biosystems, Foster City, CA, USA) according to the manufacturer’s protocol.

The selection of polymorphisms to study (rs3736729 (*GCLC*), rs207454 (*XDH*) and rs1052133 (*OGG1*)) was based on previous data from our group that has associated genetic variants of these polymorphisms with a predisposition to breast cancer.

### 3.3. Statistical Analyses

The SPSS statistical package (version 17.0) was used to analyze, by regression methods, the association between polymorphisms and clinical variables. In addition to the genetic information, the following characteristics of the patients were included in the analysis: age, hormonal receptor status (ER and PGR), Her-2 overexpression, menopausal status, histological status, grade, tumor size, nodule status, metastases, type of treatment (adjuvant or neoadjuvant), drug of treatment (anthracyclines, tamoxifen and others) and radiotherapy. The non-parametric Kruskal–Wallis method was used for testing the association between quantitative variables and polymorphisms genotypes (Table 3). The Cox regression analysis was used to adjust by variables. Imputation of missing values was performed by multiple regression. Disease-free survival (DFS) and overall survival (OS) curves were plotted according to the Kaplan-Meier method, and differences between groups were assessed using the log-rank test. DFS was defined as time (months) from diagnostic to earliest recurrence or to last contact with the patient, while OS was defined as time (months) from diagnosis to death caused by breast cancer. Other causes of death were considered as censored observations and also contribute to estimate the survival. Survival analysis was carried out for the complete patient sample, as well as subgroups, according to ER and PR status. The follow up times were censored at 10 years. In all tests, *p*-values equal to or lower than 0.05 were considered statistically significant.

## 4. Conclusions

This one is the first study that shows a relationship between polymorphisms on the *GCLC* and *XDH* genes and the survival outcome in breast cancer patients. The results obtained seem to sustain the hypothesis of the participation of oxidant and antioxidant genes in breast cancer. Unfavorable genetic variants in the rs207454 (*XDH*) and rs3736729 (*GCLC*) polymorphisms may act as predictors of outcome in negative progesterone receptor and negative estrogen receptor breast cancer patients, respectively. Further studies were necessary to confirm this result in independent population groups.

## Figures and Tables

**Table 1:** Characteristics of breast cancer patients (*n* = 470).

*n* = 470			

	Median	Range	

Age (years)	53	(20–86)	
Follow up * (months)	52.44	(0–120)	

		cases	%
		
**Menopause status**	Premenopausal	77	28.00
	Postmenopausal	187	68.00
	Perimenopausal	11	4.00

**Histologic Type**	Ductal infiltrate	317	69.06
	Inflammatory	69	15.03
	CDI intraductal	46	10.02
	Others	27	5.88

**Tumor size**	T0	7	1.65
	T1	109	25.65
	T2	205	48.24
	T3	37	8.71
	T4	67	15.9

**Node stage**	N0	153	34.77
	N1	275	62.5
	N2	9	2.05
	N3-N9	3	0.7

**Metastasis diagnosis**	M0	425	96.59
	M1	15	3.41

**Metastasis relapse**	Primary	136	28.4
	Secondary	64	13.6

**Stage**	I	53	21.9
	II	136	56.2
	III	53	21.9

**ER status**	−	219	46.79
	+	249	53.21

**PGR status**	−	239	50.96
	+	230	49.04

**HER2 overexpression**	−	195	84.78
	+	35	15.22

**Drug treatment**	Anthracyclines	159	69.74
	Tamoxifen	45	19.74
	Others	24	10.53

**Administration Treatment**	Adjuvant	326	72.4
	Neoadjuvant	99	22.00
	No treatment	25	5.56

**Radiotherapy**	No	294	62.55
	Yes	153	32.55
	NA	23	4.90

* Censured at 120 months; NA: not available; T4: not measurable inflammatory breast cancer.

**Table 2:** Analysis of disease-free survival (DFS) and overall survival in the global breast cancer population and in the subgroups of hormonal receptors by rs3736729 (*GCLC*) genotypes.

			Genotype Overall				ER+				ER−				PGR+				PGR−				ER− and PGR−		

			months	(95% CI)	*p*		months	(95% CI)	*p*		months	(95% CI)	*p*		months	(95% CI)	*p*		months	(95% CI)	*p*		months	(95% CI)	*p*
**DFS**	AA	*n* = 402	90.74	(82.74–98.74)	**0.015**	*n* = 215	91.29	(81.57–101.02)	0.887	*n* = 185	90.1	(76.26–103.94)	**0.006**	*n* = 196	97.96	(88.65–107.27)	0.111	*n* = 205	81.51	(68.10–94.93)	0.095	*n* = 150	87.64	(71.58–103.7)	**0.018**
	AC		78.62	(71.07–86.17)			92.03	(83.09–100.97)			65.01	(53.59–76.43)			94.26	(85.06–103.47)			64.82	(53.97–75.67)			60.49	(47.96–73.02)	
	CC		93.24	(83.98–102.49)			95.11	(82.86–107.36)			89.36	(74.83–103.88)			108.65	(99.69–117.61)			78.42	(63.78–93.07)			85.4	(67.92–102.89)	
						
	AA/CC		91.77	(85.71–97.82)	**0.004**		92.82	(85.23–100.41)	0.64		89.63	(79.59–99.68)	**0.001**		102.05	(95.30–108.80)	0.095		80.00	(70.08–89.92)	**0.032**		86.4	(74.51–98.30)	**0.005**
	AC		78.62	(71.07–86.17)			92.03	(83.09–100.97)			65.01	(53.59–76.43)			94.26	(85.06–103.47)			64.82	(53.97–75.67)			60.49	(47.96–73.02)	
						
	AA/AC		83.92	(78.37–89.47)	0.114		91.79	(85.19–98.38)	0.742		73.88	(64.77–83.00)	0.091		96.13	(89.60–102.67)	0.06		71.1	(62.53–79.67)	0.421		69.54	(59.28–79.80)	0.133
	CC		93.24	(83.98–102.49)			95.11	(82.86–107.36)			89.36	(74.83–103.88)			108.65	(99.69–117.61)			78.42	(63.78–93.07)			85.4	(67.92–102.89)	
						
	CC/AC		83.81	(77.88–89.75)	0.126		93.09	(85.86–100.32)	0.855		73.02	(63.75–82.29)	0.068		99.59	(92.78–106.39)	0.996		69.51	(60.74–78.29)	0.128		68.14	(57.67–78.61)	0.093
	AA		90.74	(82.74–98.74)			91.29	(81.57–101.02)			90.1	(76.26–103.94)			97.96	(88.65–107.27)			81.51	(68.10–94.93)			87.64	(71.58–103.7)	

**OS**	AA	*n* = 409	100.73	(93.74–107.72)	0.12	*n* = 218	99.00	(90.07–107.94)	0.381	*n* = 189	103.82	(92.73–114.92)	**0.037**	*n* = 199	106.84	(99.15–114.53)	0.309	*n* = 209	92.75	(80.48–105.02)	0.238	*n* = 154	102.62	(89.83–115.41)	0.052
	AC		93.02	(85.77–100.26)			102.85	(94.67–111.03)			82.99	(71.32–94.66)			104.93	(96.69–113.18)			81.91	(70.69–93.12)			80.18	(67.04–93.31)	
	CC		104.15	(96.24–112.06)			107.54	(98.17–116.90)			99.42	(85.9–112.95)			113.35	(106.05–120.65)			94.47	(80.80–108.13)			98.81	(82.48–115.15)	
						
	AA/CC		102.12	(96.87–107.37)	0.05		102.85	(94.67–111.03)	0.918		101.63	(92.90–110.36)	**0.012**		109.39	(103.89–114.90)	0.457		93.49	(84.35–102.63)	0.091		100.69	(90.38–111.00)	**0.017**
	AC		93.02	(85.77–100.26)							82.99	(71.32–94.66)			104.93	(96.69–113.18)			81.91	(70.69–93.12)			80.18	(67.04–93.31)	
						
	AA/AC		96.37	(91.25–101.48)	0.136		100.94	(94.89–106.99)	0.23		90.55	(81.93–99.18)	0.398		105.83	(100.18–111.48)	0.126		86.03	(77.61–94.45)	0.361		87.96	(78.09–97.84)	0.357
	CC		104.15	(96.24–112.06)			107.54	(98.17–116.90)			99.42	(85.90–112.95)			113.35	(106.05–120.65)			94.47	(80.80–108.13)			98.81	(82.48–115.15)	
						
	CC/AC		97.23	(91.79–102.67)	0.527		104.62	(98.39–110.84)	0.246		88.55	(79.48–97.62)	0.06		108.25	(102.44–114.07)	0.527		86.53	(77.80–95.26)	0.357		86.22	(75.77–96.67)	0.087
	AA		100.73	(93.74–107.72)			99.00	(90.07–107.94)			103.82	(92.73–114.92)			106.84	(99.15–114.53)			92.75	(80.48–105.02)			102.62	(89.83–115.41)	

Significant *p*-values are in bold.

**Table 3:** Kruskal–Wallis test of association for rs1052133, r207454 and rs3736729 with the quantitative variables age, estrogen receptor and progesterone receptor. The distribution of the variables between the genotypes is not significantly different.

	rs1052133			rs207454			rs3736729		

Variables	AA	AC	CC	AA	AC	CC	AA	AC	CC
			
Age	0.147			0.227			0.593		
Estrogen receptor	0.592			0.201			0.287		
Progesterone receptor	0.199			0.538			0.499		
